# Extracellular VirB5 Enhances T-DNA Transfer from *Agrobacterium* to the Host Plant

**DOI:** 10.1371/journal.pone.0025578

**Published:** 2011-10-18

**Authors:** Benoît Lacroix, Vitaly Citovsky

**Affiliations:** Department of Biochemistry and Cell Biology, State University of New York, Stony Brook, New York, United States of America; University of Leeds, United Kingdom

## Abstract

VirB5 is a type 4 secretion system protein of *Agrobacterium* located on the surface of the bacterial cell. This localization pattern suggests a function for VirB5 which is beyond its known role in biogenesis and/or stabilization of the T-pilus and which may involve early interactions between *Agrobacterium* and the host cell. Here, we identify VirB5 as the first *Agrobacterium* virulence protein that can enhance infectivity extracellularly. Specifically, we show that elevating the amounts of the extracellular VirB5—by exogenous addition of the purified protein, its overexpression in the bacterium, or transgenic expression in and secretion out of the host cell—enhances the efficiency the *Agrobacterium*-mediated T-DNA transfer, as measured by transient expression of genes contained on the transferred T-DNA molecule. Importantly, the exogenous VirB5 enhanced transient T-DNA expression in sugar beet, a major crop recalcitrant to genetic manipulation. Increasing the pool of the extracellular VirB5 did not complement an *Agrobacterium virB5* mutant, suggesting a dual function for VirB5: in the bacterium and at the bacterium-host cell interface. Consistent with this idea, VirB5 expressed in the host cell, but not secreted, had no effect on the transformation efficiency. That the increase in T-DNA expression promoted by the exogenous VirB5 was not due to its effects on bacterial growth, virulence gene induction, bacterial attachment to plant tissue, or host cell defense response suggests that VirB5 participates in the early steps of the T-DNA transfer to the plant cell.

## Introduction

*Agrobacterium tumefaciens* is a phytopathogenic bacterium that “genetically engineers” its hosts by transferring T-DNA from the Ti plasmid to the host cell [1], [2], [3]. Whereas, in nature, *Agrobacterium* infects plants, causing neoplastic growths at the sites of infection, under laboratory conditions, this pathogen can genetically transform eukaryotic cells from diverse origins, from plant to yeast [4], [5] to human [6], indicating the conserved nature of the transformation process [7]. This transformation represents the only known natural example of trans-kingdom DNA transfer, making *Agrobacterium* not only a tool-of-choice to produce transgenic plants for research and biotechnology, but also a unique experimental system to study general aspects of interactions between pathogenic bacteria and their eukaryotic hosts.

The translocation of T-DNA and bacterial effector proteins into host cells relies on a bacterial conjugation-related mechanism via the type 4 secretion system (T4SS) [8], [9], [10]. Whereas a substantial body of evidence suggests potential functions for many of the T4SS proteins [9], their roles in the interaction with and T-DNA transport to the host cell remain largely obscure. To begin exploring these roles, we focused on one of the exported T4SS proteins, VirB5, the function of which outside of the bacterial cell is unknown.

VirB5 is an essential virulence (Vir) protein [11] and represents a minor component of the T-pilus [12]. VirB5 interacts with VirB2 and is detected at the bacterial cell surface and at the tip of the T-pilus [13]. This secretion of VirB5 depends on other T4SS components [12]. In the bacterium, VirB5 likely participates in the biogenesis and/or stabilization of the T-pilus as *Agrobacterium* harboring a C-terminal deletion mutant of VirB5, which is unable to interact with VirB2, produces shorter pili [13]. In addition, VirB5 interacts with the bacterial trans-Zeatin biosynthetic enzyme Tzs and mediates its translocation to the cell surface [14]. Structure of the VirB5 homolog TraC was determined [15] and predicted to be similar to that of *Helicobacter pylori* CagL [16], which interacts with the T4SS pilus and binds to the host cell integrin receptor to facilitate translocation of effector proteins [17].

That VirB5 is translocated to the physical interface between the bacterial and plant cell surfaces suggests an additional role during the early interactions between *Agrobacterium* and the host cell. Among several possible types of such interactions, we identified one, the T-DNA transfer, which is affected by increased amounts of extracellular VirB5 in tobacco and sugar beet, a poorly transformable commercial crop.

## Results

### Extracellular VirB5 does not affect *Agrobacterium* cell growth, *vir* gene expression, attachment to host cells, or host defense response

**Figure 1:**
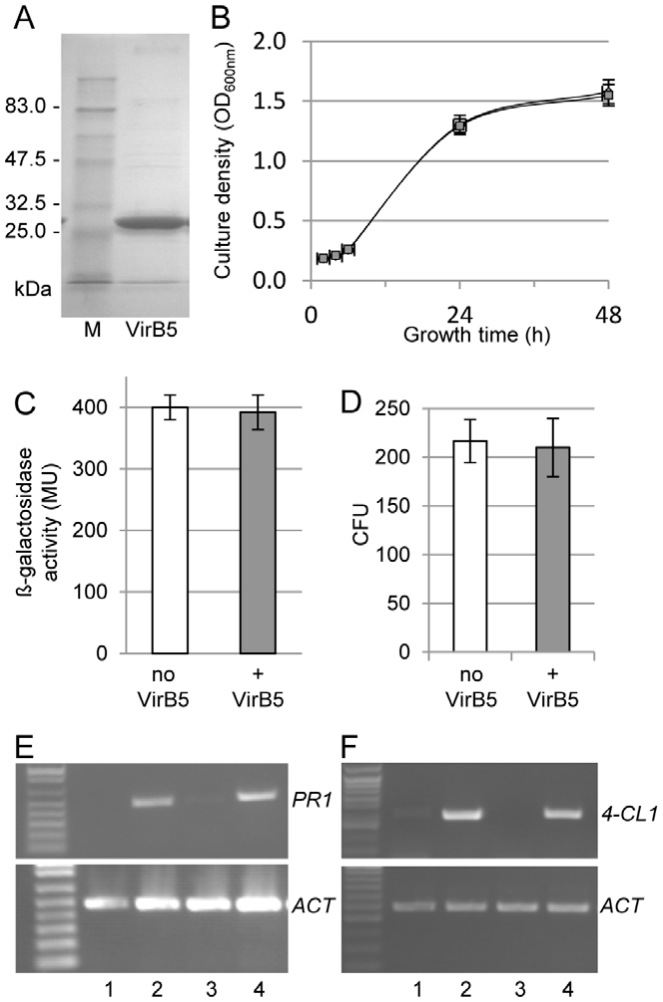
*Agrobacterium* cell growth, *vir* gene expression, attachment to host cells, and host defense response in the presence of exogenous VirB5. (A) SDS PAGE analysis of purified VirB5. (B) Growth curve of *Agrobacterium*. Gray squares, no VirB5; black squares, +VirB5. (C) *vir* gene induction by acetosyringone. MU, Miller units. (D) *Agrobacterium* attachment to plant tissues. CFU, colony-forming units. All data represent average values of three independent experiments with indicated standard deviations. Results obtained in VirB5-treated samples were not statistically different from untreated controls (*P*-values>0.2). (E) RT-PCR analysis of induction of *PR1* gene expression by treatment with salicylic acid. Lane 1, control; lane 2, +salicylic acid; lane 3, +VirB5; lane 4, +VirB5+salicylic acid. (E) RT-PCR analysis of induction of *4-CL1* gene expression by treatment with flagellin 22. Lane 1, control; lane 2, +flagellin 22; lane 3, +VirB5; lane 4, +VirB5+flagellin 22. Constitutively expressed *ACTIN (ACT)* was used as internal control (lower panels).

If extracellular VirB5 plays a role in *Agrobacterium* infection which is beyond its known intracellular functions, it might do so via five major mechanisms that affect early stages of the transformation process: bacterial cell growth, virulence (*vir*) gene induction, binding to the host cells, suppression of host defense, and enhancement of the T-DNA transfer. Because our current understanding of VirB5 provides no indication for which of these functions is associated with this protein, we tested all of them using a recombinant VirB5 purified to near homogeneity (Fig. 1A).

Fig. 1B shows that exogenous VirB5 had no effect on the bacterial growth kinetics. Similarly, induction of *vir* gene expression, assayed in an *Agrobacterium* strain containing the β-galactosidase reporter gene under the control of the *virH* promoter [18], [19], was not affected by the exogenous VirB5 in the extracellular medium (Fig. 1C).

Then, we examined whether exogenous VirB5 can alter attachment of *Agrobacterium* cells to the surface of the host plant cell. To test this hypothesis, we used a bacterial attachment assay in which the quantity of bacteria bound to plant tissues was estimated by counting colony forming units. Fig. 1D shows that no differences in *Agrobacterium* attachment to plant tissues were detected in the presence and absence of the extracellular VirB5.

Next, we measured the potential effect of additional extracellular VirB5 on plant defense induction. Tobacco protoplasts were treated with two known defense response elicitors, salicylic acid and flagellin 22 [20], [21], and the resulting transcriptional activation of the corresponding defense-related genes, *PR1* [20] and *4-CL1* [22], respectively, was monitored by RT-PCR. Fig. 1E–G shows that exogenous VirB5 had no effect on any of these defense reactions. Indeed, quantification and subsequent statistical analysis detected no significant differences between the VirB5-treated and untreated. Specifically, the Student's *t*-test yielded *P*-values>0.2 based on 108.58% of control, SD = 11.79 for *PR1* expression and 93.07% of control, SD = 12.07 for *4CL1* expression.

### Extracellular VirB5 enhances T-DNA transfer

The last potential function of the extracellular VirB5 that we tested is facilitation of T-DNA transfer, which can be estimated from the increase in the degree of transient T-DNA expression, i.e., expression of the transferred transgene before the integration event. Thus, we examined the ability of extracellular VirB5 to affect transient T-DNA expression in plant tissues. Tobacco tissues were infected with *Agrobacterium* strain, EHA105, harboring either a binary plasmid pPZP-DsRed2 with the DsRed2 marker in its T-DNA for agroinfiltration inoculations or a binary plasmid pPZP-GUSintron with the ß-glucuronidase (GUS) marker in its T-DNA for leaf disc or hypocotyl segment inoculations; T-DNA expression was quantified based on the number of cells per unit of tissue surface that express DsRed2 fluorescent signal or histochemical GUS staining, respectively.

**Figure 2:**
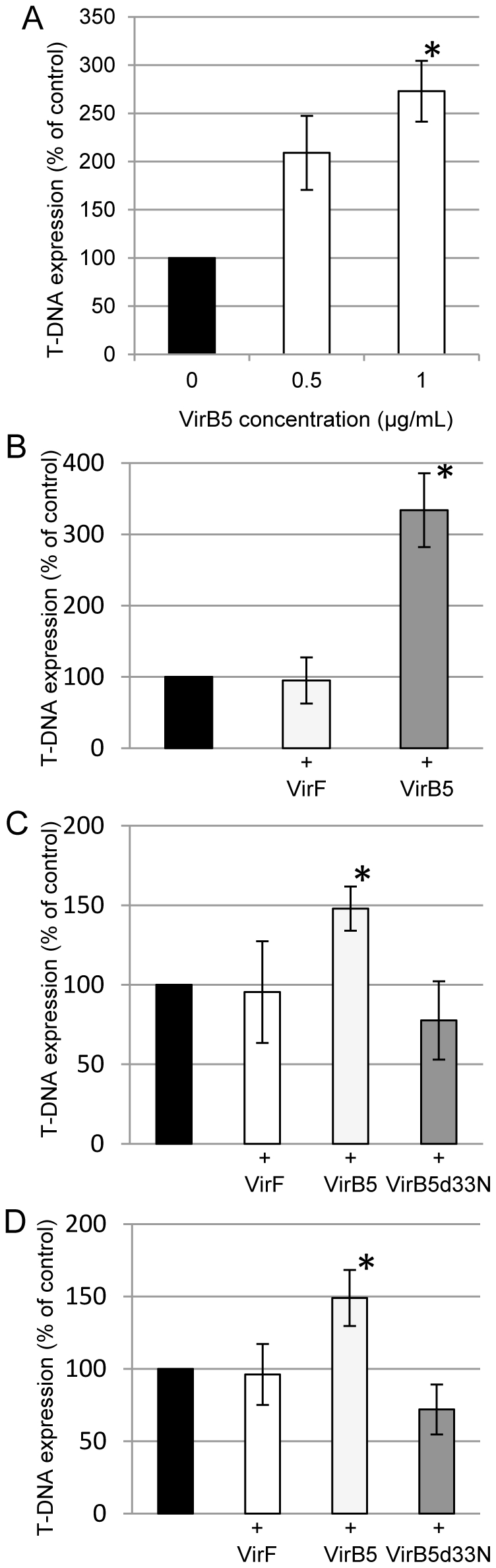
Effect of exogenous VirB5 on transient T-DNA expression. (A) VirB5 concentration effect in agroinfiltrated tobacco leaves. (B) Agroinfiltration of tobacco leaves. (C) Tobacco leaf disc inoculation. (D) Sugar beet hypocotyl segment inoculation. T-DNA control expression levels in the absence of exogenous proteins (black bars) were set at 100%. All data represent average values of three independent experiments with indicated standard deviations. Asterisks indicate a statistical significance (*P*-values<0.01 for panels A and B, and 0.05 for panels C and D) of the difference between experimental and control data.

Fig. 2 shows that the presence of exogenous VirB5 in the inoculation mixture resulted in T-DNA expression levels higher, by 50–250%, than those measured in the absence of the added VirB5. This effect was dose-dependent, with higher amount of the added VirB5 producing higher levels of T-DNA expression (Fig. 2A). The extent of this T-DNA transfer-enhancing effect of VirB5 depended on the inoculation method, being higher for agroinfiltration (Fig. 2B) and lower for leaf disc inoculation (Fig. 2C). This effect of VirB5 was specific because another purified Vir protein, VirF (Fig. 2B, C), and an N-terminal deletion mutant of VirB5, VirB5d33N (Fig. 2C), had no effect on T-DNA expression.

We then tested whether exogenous VirB5 can improve T-DNA transfer to sugar beet, an agronomically-important crop which is notoriously difficult to transform [23]. Fig. 2D shows that extracellular VirB5 substantially, by 30–70%, elevated the transient T-DNA expression levels in the inoculated sugar beet hypocotyl segments. No such effect was observed with negative controls, VirF and VirB5d33N (Fig. 2D).

### Uncoupling between the intracellular and extracellular functions of VirB5

**Figure 3:**
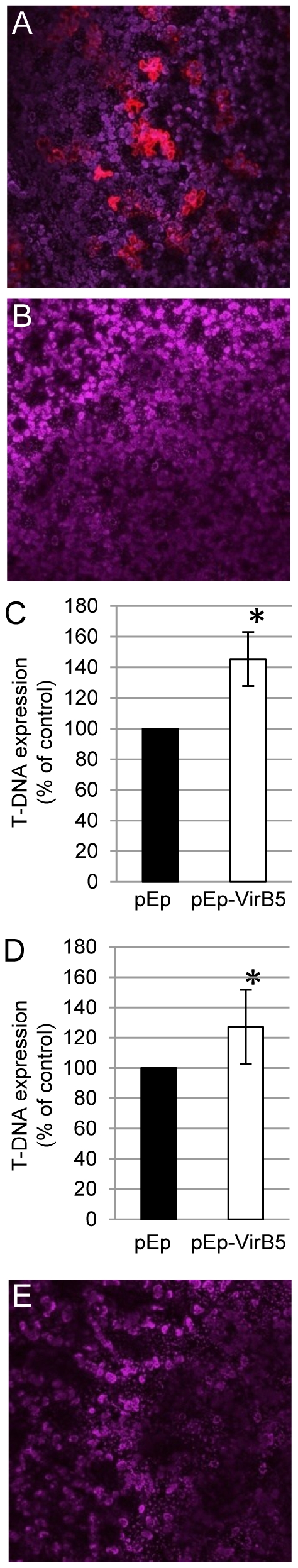
VirB5 overexpressed in *Agrobacterium*, but not exogenous VirB5, complements a mutation in the *virB5* gene. (A) Agroinfiltration of tobacco leaves with CB1005+pEp-VirB5. (B) Agroinfiltration of tobacco leaves with CB1005+pEp. (C) Inoculation of tobacco leaf discs with EHA105+pEp-VirB5 or EHA105+pEp. (D) Inoculation of sugar beet hypocotyl segments with EHA105+pEp-VirB5 or EHA105+pEp. Control expression levels in the presence of pEp (black bars) were set at 100%. All data represent average values of three independent experiments with indicated standard deviations. Asterisks indicate a statistical significance (*P*-values<0.05) of the difference between the experimental and control data. (E) Agroinfiltration of tobacco leaves with CB1005+exogenous VirB5. DsRed2 is in red, plastid autofluorescence is in purple. All images are single confocal sections.

It is important to determine whether the exogenous VirB5 in fact functions extracellularly or whether it enters the bacterial cell and acts there. To address this question, we first investigated the effects of the elevated intracellular levels of VirB5 on T-DNA transfer efficiency. To this end, *virB5* was expressed from the strongest acetosyringone-induced *vir* gene promoter, the *virE* promoter, which is approximately four times more efficient than the *virB* promoter [19]. This overexpressed VirB5 complemented a mutant *Agrobacterium* strain, CB1005 [12], deficient in *virB5*. Fig. 3A shows that inoculation with CB1005, harboring the VirB5-overexpressing plasmid pEp-VirB5 and the marker transgene plasmid pPZP-DsRed2, resulted in efficient T-DNA expression in the agroinfiltrated leaf tissues. No such expression, and therefore VirB5 complementation, was observed without VirB5 expression when the empty pEp vector was used (Fig. 3B).

Because the endogenous VirB5 is translocated to the surface of the bacterial cell, the elevated intrabacterial levels of VirB5 are expected to enhance infectivity of an *Agrobacterium* strain, EHA105, carrying the wild-type *virB5*. Indeed, Fig. 3C, D shows that *Agrobacterium* cells harboring pEp-VirB5 and pPZP-GUSintron induced T-DNA expression to levels consistently higher, by 20–60%, than those of EHA105 carrying the control pEp plasmid and pPZP-GUSintron. This ability of overexpressed VirB5 to improve *Agrobacterium* infectivity was observed both in tobacco leaf discs (Fig. 3C) and in sugar beet hypocotyls (Fig. 3D). Furthermore, following secretion, VirB5 overexpressed in *Agrobacterium* lacks its signal peptide [24], whereas the exogenously added full-length VirB5 retains this sequence; thus, the VirB5 signal peptide most likely is not involved in the extracellular function of this protein.

Finally, we examined whether the exogenous VirB5 can complement the *virB5* mutation in CB1005. Fig. 3E shows that, when VirB5 was added to the agroinfiltration mixture, CB1005 remained completely unable to promote T-DNA expression. That the extracellular VirB5 is unable to complement the *virB5* mutation in *Agrobacterium* while capable of enhancing infectivity of the bacterial strain with the wild-type *virB5* (see Fig. 2) suggests a dual function for VirB5: one within the *Agrobacterium* and another at the bacterium-host cell interface.

### Secretion of recombinant VirB5 by the host cell enhances T-DNA transfer

**Figure 4:**
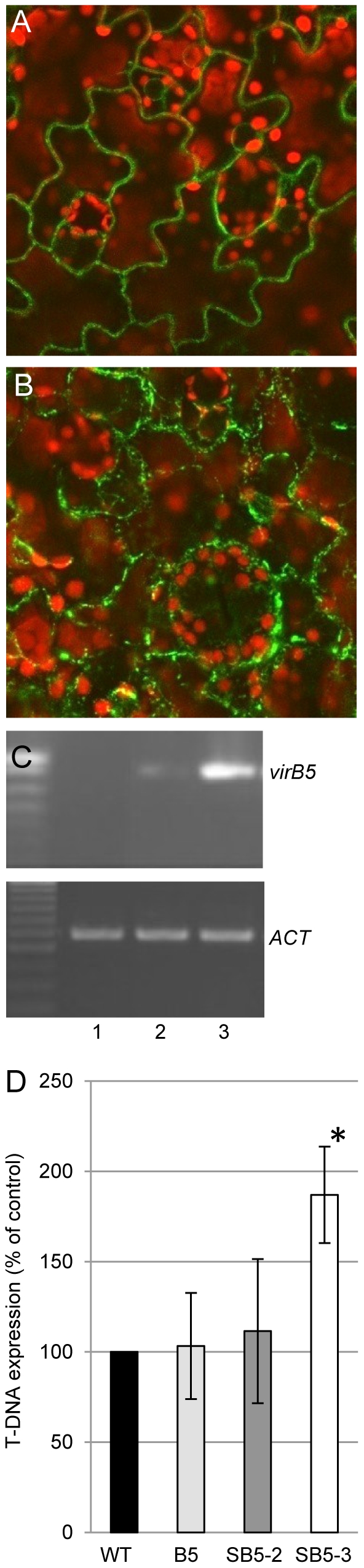
Effect of VirB5 secreted from transgenic plants on T-DNA expression. (A) Subcellular localization of GFP-secVirB5 in tobacco leaf cells. (B) Subcellular localization of GFP-secVirB5 in plasmolysed tobacco leaf cells. GFP is in green, plastid autofluorescence is in red. All images are single confocal sections. (C) RT-PCR analysis of sec*virB5* gene expression in low and high expresser transgenic lines. Lane 1, wild-type plant; lane 2, SB5-2; lane 3, SB5-3. Constitutively expressed *ACTIN (ACT)* was used as internal control (lower panel). (D) T-DNA expression in inoculated leaf discs from the SB5-2 and SB5-3 lines that express secVirB5, and from the B5 line that expresses VirB5. Control expression level in the wild-type plants (WT, black bar) was set at 100%. All data represent average values of three independent experiments with indicated standard deviations. Asterisk indicates a statistical significance (*P*-value<0.05) of the difference between the experimental and control data.

Next, we examined whether the extracellular VirB5 may exert its effect on the efficiency of T-DNA transfer inside the host cell, for example, after entering its cytoplasm. To this end, we constructed transgenic tobacco plants that express the full-length VirB5 either intact or fused to a eukaryotic N-terminal secretion signal (secVirB5) or tagged with GFP and fused to the secretion signal (secGFP-VirB5). These transgenic plants showed no visible developmental or morphological phenotypes, indicating that VirB5 does not interfere with vital cellular functions. Next, we examined whether secGFP-VirB5 is indeed secreted from the expressing cells. Fig. 4A shows that secGFP-VirB5 localized mainly at the cell periphery. We distinguished plasma membrane targeting from cell wall localization by plasmolysis experiments [25], where the secGFP-VirB5 fluorescent signal associated with the cell wall, but not with the displaced membrane in plasmolysed cells (Fig. 4B), indicating protein secretion.

We then analyzed the effects of plant-secreted secVirB5 and plant intracellular VirB5 on T-DNA expression using the EHA105 strain harboring pPZP-GUSintron. For secVirB5, we selected two transgenic lines, a high and a low expresser (Fig. 4C). Quantification of the PCR data in Fig. 4C indicated statistical significance of the difference between these expression levels as determined by Student's *t*-test with *P*-value<0.01 based on 14.33 arbitrary units (AU) with SD = 8.39 for the SB5-2 line and 86.00 AU with SD = 10.44 for the SB5-3 line. Fig. 4D shows that the high expresser line SB5-3 substantially increased T-DNA expression as compared to the wild-type plants. This effect was secVirB5 dose-dependent because the T-DNA expression levels in the low expresser SB5-2 line showed no statistically significant difference from the wild-type plants (Fig. 4D). Importantly, VirB5 without the plant secretion signal expressed in transgenic line B5 had no effect on T-DNA expression (Fig. 4D). Thus, VirB5 acts extracellularly, but has no function within the host cell. The extracellular function of VirB5 is independent of its origin, whether from the bacterial or the host cell.

## Discussion

Our data suggest that VirB5 plays a dual role in the genetic transformation process. Its first role, which involves participation in the formation and stabilization of the T4SS macromolecular complex and particularly the T-pilus, is well known [12], [13]. The second role, which is likely played at the interface between the bacterial and the plant cells to enhance translocation of the T-DNA and/or effector proteins, is demonstrated here for the first time. The proposed ability of VirB5 to enhance T-DNA transfer is consistent with its predicted structural homology to the *H. pylori* CagL, which facilitates macromolecular transport into the host cell [17]. This extracellular function of VirB5 occurs independently of the bacterial binding to the host cell, growth rate, induction of the virulence system, or suppression of the host defense. These two functions of VirB5 are distinct from each other because the extracellular activity does not complement that of VirB5 expressed within the bacterial cell. Furthermore, VirB5 can fulfill its function irrespective of the mechanism by which this protein had reached its destination: export from *Agrobacterium*, exogenous addition, or export from the host cell, indicating that this VirB5 activity occurs at the extracellular site and not en route to this location. The extracellular role of VirB5 is supported by earlier studies of its homolog TraC involved in bacterial conjugation in *E. coli* which showed that a TraC mutant defective for conjugation could be partially complemented by coculturing with another bacterial strain that produced active TraC [26]. Furthermore, similarly to VirB5, TraC was found to localize to the surface of the bacterial cell and associate with extracellular structures in *E. coli* [27]. Obviously, because VirB5 functions in macromolecule transport between *Agrobacterium* and host plants, rather than in bacterial conjugation, it may have evolved additional functions as compared to TraC.

Another important aspect of our findings is that they identify the first T4SS component, or any other *Agrobacterium* Vir protein, that can enhance infectivity when simply added in its purified form to the inoculation mixture. This demonstrates that the function of some T4SS proteins is set not only during the biogenesis of the secretion apparatus, but also can be effected when the protein is supplied exogenously. Because T4SS is employed by such mammalian pathogens as *Bartonella henselae* [28], *Brucella melitensis*, and *Legionella pneumophila* [8], [10], it is tempting to speculate that their VirB5 homologs can serve as good targets for simple pharmaceutical intervention to block their interactions with the host cells.

The third significant aspect of our data is their implication for plant biotechnology. Exogenously added VirB5 enhanced T-DNA transfer and transient T-DNA expression in sugar beet, a major crop recalcitrant to *Agrobacterium* [23]. Our experiments focus on the effect of VirB5 on the early stages of the *Agrobacterium*-mediated genetic transformation, i.e., the T-DNA transfer to and transient expression in the host cell, rather than on subsequent T-DNA integration and stable transformation. However, because T-DNA transfer is a prerequisite for stable genetic transformation, we speculate that genetic manipulation of some agronomically-important plant species, which are difficult to transform using *Agrobacterium*, can be improved by technically simple and cost effective addition of recombinant VirB5 to the transformation reactions.

## Materials and Methods

### Constructs

The full-length octopine *virB5* (AAZ50522), including its 20 amino-acid N-terminal bacterial secretion signal, was amplified from pTiA6 using the primer pair 5′CCGGAATTCATGAAGACGACGCAACTTATTGCA3′/5′CCGCTCGAGTTAGGGGAGGGCACCAAAGAT3′ and cloned into the EcoRI-XhoI sites of pET28a(+) (Clontech). Coding sequence for VirB5d33N, which lacks 33 N-terminal amino acid residues, was produced using the same strategy and forward primer 5′CCGGAATTCATGGAGAATCTCACTCAGACTATAG3′. The pSAT5-sec plasmid for plant expression of secreted proteins was made by inserting into the NcoI-BglII sites of pSAT5 [29] the sea anemone equistatin N-terminal secretion signal amplified from ImpactVector 1.2 (Plant Research International, Wageningen) using the primer pair 5′CATGCCATGGGCTCTCTTAGCCAGAACCAG3′/5′CACAGATCTGACTAGCTTCAGTTGAAGTG3′. For pSAT5-secVirB5, *virB5* was inserted into the EcoRI-XhoI PCR sites of pSAT5-sec. For pSAT5-secGFP-VirB5, the GFP coding sequence was inserted into the BglII-EcoRI sites of pSAT-secVirB5 between the secretion signal and *virB5*. For pSAT5-VirB5, *virB5* was inserted into the EcoRI-XhoI PCR sites of pSAT5A [30]. Note that although GFP is susceptible to pH variations [25], it has been consistently and successfully used to monitor cell wall localization of secreted protein in plants [31], [32]. For agroinfiltration, all pSAT5-derived constructs were digested with the rare-cutting I-CeuI, to excise the entire expression cassette, which was then cloned into the I-CeuI site of pCB370ZP [33].

For measuring T-DNA expression, we produced two binary constructs, pPZP-DsRed2 and pPZP-GUSintron. pPZP-DsRed2 was constructed by cloning the DsRed2 expression cassette from pSAT6-DsRed2-C1 into the PI-PspI site of pRCS2-PZP [29]. The GUSintron sequence from pBISN1 [34] amplified using the primer pair 5′GGAAGATCTATGTTACGTCCTGTAGAAACCCC3′/5′CCGGAATTCTCATTGTTTGCCTCCCTGCTGS3′) was first inserted into the EcoRI-BamHI sites of pSAT5 [29], and then the entire GUSintron expression cassette was cloned into the I-CeuI site of pRCS2-*nptII* [29].

For overexpression of VirB5 in *Agrobacterium*, the *virE* promoter (*virE*p) amplified with the primer pair 5′ATGCCATGGGATAAGTCGCCAATATAGTGATC3′/5′CCGGTGACAGATCTGGTACCGGATCCGAATTCATGTTCTCTCCTGCAAAATTCG3′ was inserted into the NcoI-SalI sites of the pCB302 [33] backbone amplified using the primer pair 5′GGCGTCGACCATCATCATCATCATCACTAAGGCTCACCGGGCTGGTTG3′/5′ATGCCATGGAGTAAAGCGCTGGCTGAACCC3′, resulting in pEp with the following multiple cloning site: NcoI-*virE*p-EcoRI-BamHI-KpnI-BglII-SalI-6xHis-STOP. *virB5* was then cloned as an EcoRI-XhoI PCR-amplified fragment into the EcoRI-SalI sites of pEp, resulting in pEp-VirB5.

### *Agrobacterium* strains

Most of the experiments were performed with the EHA105 strain of *Agrobacterium tumefaciens*. The *virB5*-deficient mutant strain CB1005 [12], a derivative of C58, was a kind gift from Dr. C. Baron (Université de Montreal, Canada).

### Plants

Tobacco plants (*Nicotiana tabacum*, var. Turk) were grown either in soil or on MS medium (10 g.L^−1^ sucrose, 8 g.L^−1^ agar) after seed surface sterilization, and maintained *in vitro* by micro-cuttings on high sucrose MS medium (30 g.L^−1^ sucrose, 8 g.L^−1^ agar). Sugar beet (*Beta vulgaris*, var. saccharina) seeds were obtained from Dan Bjur (SES Vanderhave Fargo, ND) and germinated in soil or on MS medium after surface sterilization. All plants were grown in an environment-controlled growth chamber under long day (16 h light/8 h dark) conditions and at 22°C.

Tobacco transgenic plants were produced using the classical leaf disc protocol [35]. Transgenic plants were selected on MS regeneration medium (10 g.L^−1^ sucrose, 8 g.L^−1^ agar, 10 mg.L^−1^ BAP, 1 mg.L^−1^ NAA) containing 50 mg.L^−1^ timentin and 8 mg.L^−1^ BASTA, and then transferred to rooting medium with the same composition as the regeneration medium, but lacking phytohormones. Transgene expression was assayed either by RT-PCR after RNA extraction from the transgenic leaf tissues or, for secGFP-VirB5 lines, by observing GFP fluorescence under a Zeiss LSM 5 Pascal confocal laser scanning microscope.

### Preparation of recombinant VirB5

The recombinant His-tagged VirB5 or VirB5d33N were expressed from the pET28a(+) vector in BL21(DE3) *Escherichia coli* strain (Novagen) as described [36], [37], and purified on Ni-NTA resin (Qiagen). This procedure resulted in a >95% pure protein preparations as determined by SDS-polyacrylamide gels electrophoresis (PAGE) and silver staining.

### Transient T-DNA expression

Agroinfiltration was used in leaves of 4–6 weeks-old tobacco and sugar beet plants grown in soil. An overnight *Agrobacterium* culture was centrifuged and the pellet resuspended in MES buffer (10 mM MgCl_2_, 10 mM MES, pH 5.6) at OD_600 nm_ = 0.6. The cell suspension was then diluted 1∶800 in MES buffer and infiltrated into the leaves using a needleless 1-mL syringe [38]. Purified recombinant VirB5 (1 µg.mL^−1^) was added to the bacterial suspension just before infiltration. After three days, one-cm squares were excised from the infiltrated zone, excluding the infiltration point itself which is often damaged by the syringe, and the number of cells expressing the DsRed2 marker from the T-DNA in pPZP-DsRed2 was counted under a confocal microscope. To avoid variability due to potential differences in susceptibility to *Agrobacterium* between different areas of the leaf, three points of infiltration were done on each side of the main vein of each leaf, such that one side was always infiltrated with an experiment and the equivalent area of the other side - with its control. Each experiment utilized three leaves.

Leaf disc and hypocotyl segment inoculations were performed as described [39], [40], using cell cultures prepared as described above for agroinfiltration. Bacterial suspension at an OD_600 nm_ of 0.25 and 0.6 were used, for tobacco leaf discs and sugar beet hypocotyls segments, respectively. T-DNA expression was monitored using the GUS marker from pPZP-GUSintron, rather than DsRed2 as in leaf agroinfiltration, due to high autofluorescence background of the excised leaf disc tissues. For histochemical detection of GUS activity, the tissues were stained with the chromatogenic substrate X-Gluc as described [41], and the number of stained areas was counted. Each experiment was repeated at least three times, with at least nine leaf discs or twenty hypocotyl segments per experiment. For both agroinfiltration and explant inoculation experiments, we quantified the number of cell expressing DsRed and the number of GUS-stained areas because these parameters directly reflect the number of transformed cells and, thus, the T-DNA transfer efficiency, whereas measuring the global DsRed signal or GUS activity also reflects the reporter gene expression levels, which are not directly indicative of the efficiency of transformation. All quantitative data were analyzed by the one-tailed Student't *t*-test; *P*-values<0.05, corresponding to the statistical probability of greater than 95%, were considered statistically significant.

### *Agrobacterium* attachment, *vir* gene induction, and bacterial growth kinetics

Attachment of *Agrobacterium* cells to plant tissue was measured using a protocol modified from [42]. Briefly, nine tobacco leaf discs were floated on a suspension of *Agrobacterium* in MES buffer for exactly 30 min, and gently rinsed three times with 10 mL MES buffer. Then, the leaf discs were transferred to a 50-mL conical tube, containing 10 mL of MES buffer. The attached bacteria were released into the medium by vortexing, and increasing dilutions of the medium were plated on the LB solid medium, containing kanamycin. After two days of incubation at 28°C, the number of colony-forming units (CFU) was recorded, reflecting the number of bacterial cells attached to the plant tissue.

For *vir* gene induction, we used an octopine-type *Agrobacterium* strain A348 harboring the pSM219 plasmid that carries *lacZ* under the control of the *virH* promoter in *trans* to the wild-type pTiA6 plasmid [18], [19]. The reporter *β*-galactosidase activity was measured after overnight induction with 100 µM acetosyringone and expressed in Miller units calculated as described [18], [19], [43].

For *Agrobacterium* growth kinetics, an overnight suspension of the EHA105 strain was diluted to OD_600 nm_ = 0.1 in M9 minimal medium, pH 5.8, supplemented with 0.2% glucose, with or without purified VirB5 (1 µg.mL^−1^). The culture was then incubated at 28°C, 240 rpm, and the cell density (OD_600 nm_) was monitored at the indicated time points.

### Induction of defense-related gene expression by salicylic acid and flagellin 22

Salicylic acid and flagellin induction was performed in tobacco protoplasts prepared as described [44]. Protoplast suspension (1 mL in MS liquid medium containing 0.4 M mannitol and 0.5 g.L^−1^ MES pH 5.8) containing approximately 0.6×10^6^ cells was placed in 35×10-mm tissue culture dish. Salicylic acid or flagellin 22 (final concentrations 50 µM and 100 nM, respectively) was added to the protoplast suspension in the presence of the purified VirB5 or VirF (1 µg.mL^−1^). After a 6-h incubation period under light at room temperature, protoplasts were harvested, and the expression of the induced genes was analyzed by RT-PCR. For the salicylic acid induction, the NtPR1 (CAA29392) expression was measured using the primer set 5′ATGGGATTTGTTCTCTTTTCACAATTG3′/5′TTAGTATGGACTTTCGCCTCTATAATTAC3′ that amplifies the 507-bp full-length NtPR1 cDNA, and for the flagellin 22 induction, the Nt4-CL1 (NTU50845) expression was measured using the primer set 5′ATGCCAATGGAGACTACTACAG3′/5′CTCAGGTTCATGCTCATGCTCATCG3′, that amplifies a 520-bp CL1 cDNA fragment. All PCR products were quantified by scanning densitometry, and the resulting data analyzed by the one-tailed Student's *t*-test.
